# Tracing narrative development in children with speech, language, and communication difficulties: a *CiteSpace* analysis

**DOI:** 10.3389/fpsyg.2026.1838439

**Published:** 2026-09-02

**Authors:** Ruijie Zhao, Yusong Gao, Yu Cui, Xiaochen Wang

**Affiliations:** 1 School of Foreign Studies, Xi’an Jiaotong University, Xi’an, Shaanxi, China; 2 College of Foreign Languages and Literature, Northwest Normal University, Lanzhou, Gansu, China; 3 School of Foreign Languages and Literatures, Chongqing University of Education, Chongqing, China

**Keywords:** bibliometric analysis, children, CiteSpace, narrative development, speech, language, communication difficulties

## Abstract

Narrative development is central to children’s language, communication, and academic functioning, yet children with speech, language, and communication difficulties often experience challenges in organizing and expressing coherent stories. This study used CiteSpace to analyze 628 articles and reviews retrieved from the Web of Science Core Collection and published between 1985 and 2024. The publication trend was descriptively divided into three periods: the Emerging Stage, the Development Stage, and the Growth Stage. Collaboration analyses identified countries, institutions, and authors with relatively high publication output and observable collaborative links. CiteSpace generated seven keyword clusters, which were subsequently synthesized by the authors into four broader themes: Language and Communication Disorders, Cognitive and Social Dimensions of Communication, Medical and Developmental Perspectives, and Speech Rate and Communication Fluency. Keyword co-occurrence analysis identified major research hotspots, while citation burst analysis showed that prevalence and developmental language disorder were prominent recent topics. Overall, the study maps the development, thematic structure, collaboration patterns, and recent areas of scholarly attention in this field and provides directions for future bibliometric and empirical research.

## 1 Introduction

Narrative development follows a series of universal milestones, progressing from simple event descriptions to structured, coherent storytelling (Bohnacker and Gagarina, 2020; Riehl, 2013; Trabasso and Nickels, 1992). However, the development of different narrative structures does not occur in parallel and can generally be analyzed at two levels: macrostructure and microstructure (Karasinski, 2023). Macrostructure refers to the overall framework of a story, including plot structure, temporal sequencing, and causal relations. These global narrative features exhibit a high degree of cross-linguistic universality, as research indicates that children tend to follow similar organizational patterns in their storytelling (Gagarina, 2016). In contrast, microstructure pertains to the specific linguistic realizations of narratives, such as syntactic complexity, lexical diversity, and referential expressions. These language-specific elements are influenced by children’s linguistic proficiency and may vary depending on the structural characteristics of different languages (Fan and Xu, 2024). Narrative skills are commonly elicited through storytelling and retelling tasks. Storytelling involves generating a narrative from pictures, prompts, personal experiences, or imagined events, whereas retelling requires children to reconstruct a previously presented story.

The development of narrative skills is crucial for children’s overall language and communication abilities, yet children with speech, language, and communication difficulties often face significant challenges in this area (Melzi et al., 2022; Norbury and Bishop, 2003; Vandewalle et al., 2012). Narrative development involves the ability to structure, understand, and retell stories, which is critical for social interaction, academic success, and cognitive growth (Gardner-Neblett, 2022; Kawar, 2024; Plotka and Wang, 2020; Shaqiri et al., 2022). However, children with speech, language, and communication difficulties, including those with speech sound difficulties, fluency difficulties, or developmental language disorder, often experience difficulties in organizing coherent narratives, understanding story sequences, and using appropriate linguistic markers (Félix et al., 2024; Gillon et al., 2020; Jensen de López et al., 2021; McGregor, 2020). Research in this field is important for three key reasons: first, narrative skills are directly linked to broader language development and literacy, meaning that children with speech, language, and communication difficulties may face academic challenges (Paradis, 2023); second, understanding these challenges can lead to more effective interventions and therapeutic strategies (Gillam et al., 2023); and third, narrative tasks are widely used as diagnostic tools, making it essential to explore how these difficulties affect narrative ability (Al-Dabbagh et al., 2023). Given the significance of narrative development in multiple domains, a comprehensive review of the literature is crucial to consolidate findings, identify gaps, and guide future research. This review provides a clearer understanding of the current research landscape, identifies key research gaps, and highlights emerging directions that may inform future investigations into narrative development in children with speech, language, and communication difficulties.

Based on this, the study uses *CiteSpace* to systematically analyze 628 articles and reviews retrieved from the SSCI, SCIE, and AHCI indexes. By gathering and analyzing these articles, this research aims to provide a structured overview of research on narrative development in children with speech, language, and communication difficulties. Furthermore, this study seeks to address the following key questions:

*Q1*: What are the general publication trends in research on narrative development in children with speech, language, and communication difficulties?

*Q2*: What are the main research themes in this field?

*Q3*: Which countries, institutions, and authors are the major contributors to this field?

*Q4*: What are the main research hotspots in this field?

*Q5*: What are the emerging research frontiers in this field?

## 2 Research design

### 2.1 Research tool

CiteSpace is a widely used bibliometric analysis tool designed to visualize trends and patterns in scientific literature (Sun et al., 2023; Wu et al., 2021). Developed by Dr. Chaomei Chen, CiteSpace enables researchers to explore the evolution of research fields, identify key contributors, and detect emerging trends over time. CiteSpace was selected for this study because its functions are suitable for tracking research trends, identifying major contributors, and detecting emerging themes in a systematic manner. Given the interdisciplinary nature of research on narrative development in children with speech, language, and communication difficulties, which encompasses linguistics, psychology, and education, CiteSpace’s functions for analyzing citation networks, keyword co-occurrences, and research clusters are particularly relevant to the objectives of this study. Citation burst detection helps identify topics that have received rapidly increasing scholarly attention, while collaboration network analysis maps institutional and geographical contributions. These features align with the objectives of the present study, particularly the analysis of research trends, collaboration networks, thematic patterns, and emerging research frontiers.

### 2.2 Data collection

The literature search was conducted in the Web of Science Core Collection and was completed on June 18, 2024, covering all records available from database inception to that date. Three citation indexes were searched, namely the Social Sciences Citation Index, the Science Citation Index Expanded, and the Arts and Humanities Citation Index. Only English-language journal articles and review articles were included, while conference papers, book chapters, dissertations, editorials, and other document types were excluded. The complete search syntax used in the abstract field was: AB = (Children AND (Speech impairment OR Speech disorder OR Language impairment OR Language disorder OR Speech language pathology OR Communication impairment OR Communication disorder OR Communication disability) AND (Storytelling OR Retelling OR Narrative)). The disorder-related terms were selected to capture the overlapping terminology used in research on children with speech, language, and communication difficulties. The narrative-related terms were intended to cover narrative generation, reconstruction, development, comprehension, assessment, and related practices. After the database restrictions were applied, 628 articles and reviews were retrieved and included in the CiteSpace analysis. This dataset represents the literature identified from the selected Web of Science indexes rather than the entire body of research in the field.

### 2.3 Data analysis

The data analysis in this study, conducted using *CiteSpace*, employed a structured bibliometric approach to explore multiple dimensions of the research field. A time-series analysis of annual publication trends was performed to trace the evolution of research directions, revealing the field’s growth and emerging focus areas. Keyword clustering analysis, using *CiteSpace*’s log-likelihood ratio algorithm, identified core themes and dominant research topics by grouping frequently co-occurring terms. To examine geographic and institutional contributions, a co-authorship network analysis mapped collaborations among countries, institutions, and authors to describe observable collaboration patterns. Keyword co-occurrence analysis, utilizing the term frequency-inverse document frequency method, identified research hotspots by detecting frequently discussed topics and their evolving significance. Additionally, citation burst detection analysis, based on Kleinberg’s burst detection algorithm, was applied to uncover emerging research frontiers by identifying sudden increases in citation frequency, signaling shifts in scholarly attention. Together, these analyses provide a systematic bibliometric examination of publication patterns, collaboration networks, prominent thematic patterns, and emerging areas of scholarly attention in research on narrative development in children with speech, language, and communication difficulties.

## 3 Results

### 3.1 General trend

**Figure 1:**
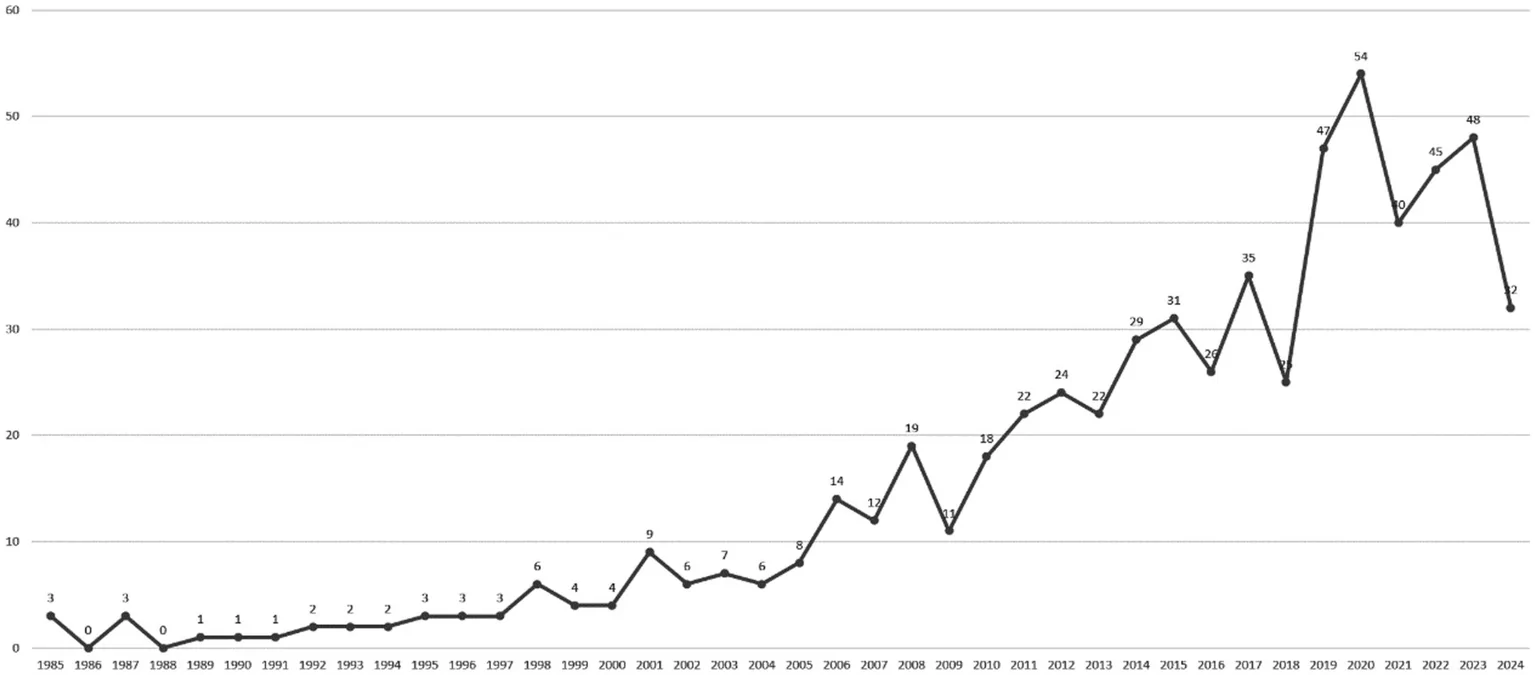
The general trend.

Figure 1 presents the annual publication output from 1985 to 2024. For descriptive purposes, the publication trend was divided into three periods: the Emerging Stage (1985–2005), the Development Stage (2006–2018), and the Growth Stage (2019–2024). These periods were identified based on visually observable changes in annual publication volume and growth patterns and should not be interpreted as statistically derived developmental stages. During the Emerging Stage, the annual number of publications remained relatively low, with only limited fluctuations. The Development Stage showed a gradual increase in publication output, although the annual growth pattern was not uniform. During the Growth Stage, the number of publications increased more noticeably, particularly in the later years. Overall, the results indicate a general upward trend in scholarly output on this topic.

### 3.2 Research themes

**Figure 2:**
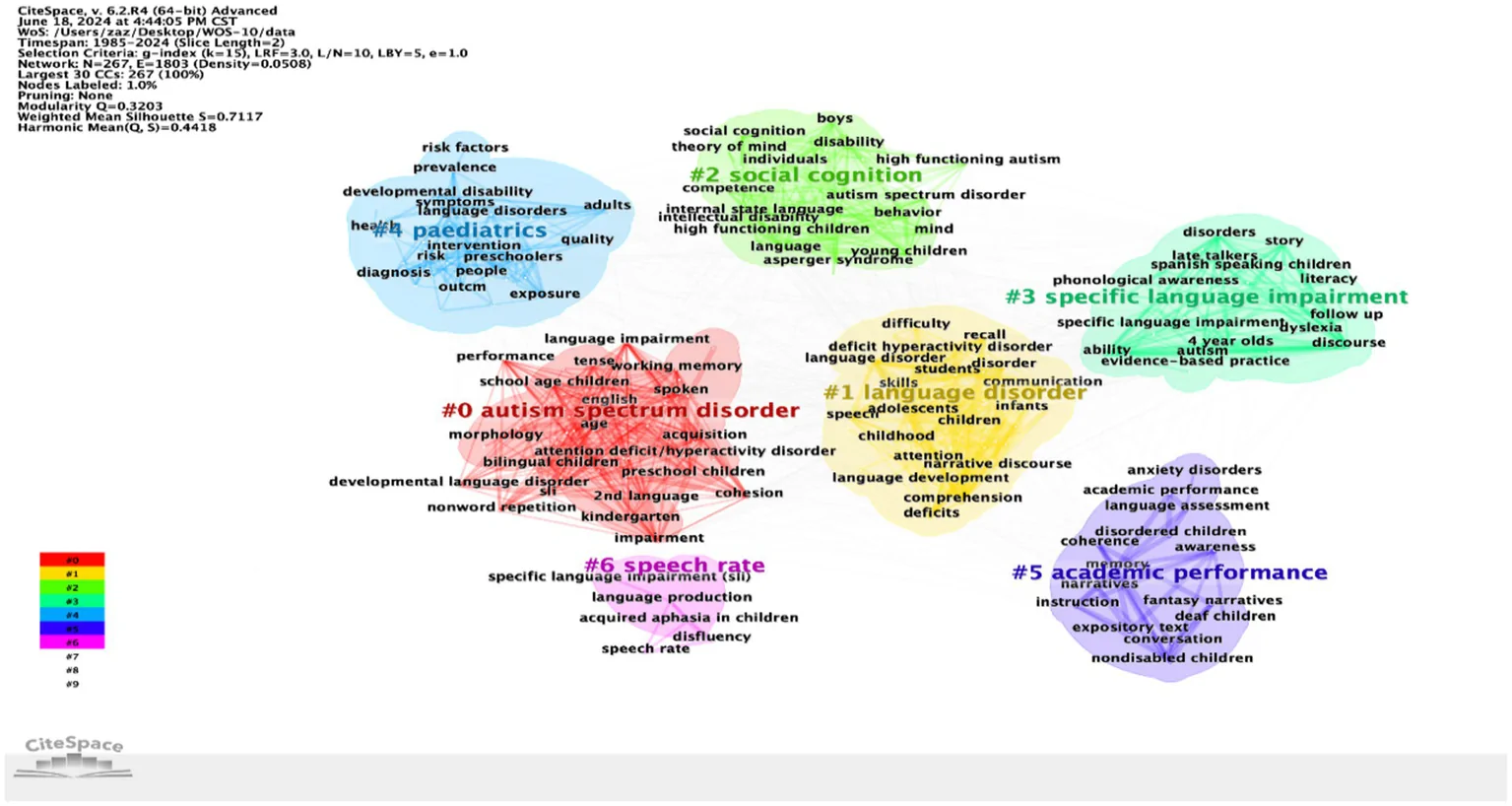
Research themes.

CiteSpace generated seven keyword clusters based on patterns of keyword co-occurrence: autism spectrum disorder (#0), language disorder (#1), social cognition (#2), specific language impairment (#3), pediatrics (#4), academic performance (#5), and speech rate (#6). These clusters and their labels constitute the direct outputs of the bibliometric analysis. To facilitate interpretation, the authors further synthesized the seven clusters into four broader research themes by examining the conceptual relationships among the cluster labels, prominent keywords, and the main research concerns represented within each cluster. The Language and Communication Disorders theme combined clusters #1 and #3; the Cognitive and Social Dimensions of Communication theme combined clusters #0 and #2; the Medical and Developmental Perspectives theme combined clusters #4 and #5; and the Speech Rate and Communication Fluency theme was derived from cluster #6. The seven clusters therefore represent CiteSpace-generated findings, whereas the four themes constitute an author-led interpretive synthesis. These groupings were used to organize related research patterns and do not imply diagnostic equivalence among speech impairments, language disorders, and autism spectrum disorder (see Figure 2).

#### 3.2.1 Language and communication disorders

This theme focuses on speech and language difficulties represented by the language disorder cluster (#1) and the specific language impairment cluster (#3). Research within these clusters examines how developmental language disorder and specific language impairment affect children’s ability to understand, produce, and organize narratives (Kohnert et al., 2020; Leonard, 2017). Common difficulties include restricted vocabulary, reduced syntactic complexity, weak cohesion, and problems maintaining a logical sequence of events in storytelling (Boschi et al., 2017; Ploog et al., 2013). Specific language impairment is an earlier term that remains visible in the literature, whereas developmental language disorder is now more widely used to describe persistent language difficulties that cannot be explained by an obvious biomedical condition. Studies also consider how these language-related challenges influence broader communication and social participation (Boucher, 2012). By synthesizing clusters #1 and #3, this theme highlights a shared focus on the relationship between language difficulties and narrative competence, while recognizing that the terminology and diagnostic criteria have evolved across the literature (Matson and Neal, 2010).

#### 3.2.2 Cognitive and social dimensions of communication

This theme integrates the autism spectrum disorder cluster (#0) and the social cognition cluster (#2), both of which address the cognitive and interpersonal processes involved in narrative communication. Children with autism spectrum disorder may experience difficulties in perspective taking, emotion understanding, and social communication, all of which can influence the coherence and listener orientation of storytelling (Kasari and Smith, 2013; Keen et al., 2016). Social cognition enables children to interpret social cues, represent characters’ intentions, and construct narratives that are meaningful to others (Didehbani et al., 2016). Research in these clusters therefore examines how cognitive and social processes shape narrative organization, relevance, and communicative effectiveness (Mundy, 2018). Autism spectrum disorder is not treated here as equivalent to a speech or language disorder. Rather, autism-related studies are included because narrative performance is closely connected to social communication and social-cognitive functioning. The theme also includes research on interventions designed to strengthen social communication and narrative abilities through structured and targeted support (Fuller and Kaiser, 2020; Scassellati et al., 2018).

#### 3.2.3 Medical and developmental perspectives

This theme combines the pediatrics cluster (#4) and the academic performance cluster (#5) to capture the broader developmental, clinical, and educational significance of children’s narrative abilities. Research in the pediatrics cluster addresses early identification, developmental monitoring, and professional support for children with speech and language difficulties (Shekerdemian et al., 2020; Thornton et al., 2015). It also considers developmental milestones in language acquisition and medical or developmental conditions that may affect narrative production (Bird et al., 2016; Kaiser et al., 2015). The academic performance cluster focuses more directly on the relationships among language ability, narrative competence, literacy development, and educational outcomes. Studies represented in these clusters also examine the contribution of speech-language therapy and other forms of professional support to children’s communication development (Bosgraaf et al., 2020; Grolig et al., 2020; Scharff-Rethfeldt et al., 2020). Their synthesis reflects a shared concern with how narrative skills develop across clinical and educational contexts, while the two clusters remain distinct outputs of the CiteSpace analysis (Cochran et al., 2023; Noble et al., 2020).

#### 3.2.4 Speech rate and communication fluency

This theme corresponds to the speech rate cluster (#6) and focuses on the temporal and fluency-related characteristics of children’s narrative production. Research in this cluster examines how the speed, rhythm, and continuity of speech influence narrative clarity and communicative effectiveness. Children with speech impairments may experience difficulty regulating speech rate, which can affect their ability to produce coherent and appropriately paced stories (Andreou et al., 2024; Egbe et al., 2023; Grønbæk et al., 2021). Speech that is excessively rapid may reduce intelligibility or disrupt narrative organization, whereas unusually slow speech may affect fluency and listener engagement (Braden et al., 2021). Studies also investigate developmental and physiological factors associated with speech rate, including motor coordination, articulatory control, and cognitive processing speed (Bhat, 2020; Firoozehchi et al., 2023). By examining these temporal features, researchers seek to clarify how speech rate contributes to narrative performance and to inform support aimed at improving fluency, pacing, and overall communication effectiveness (Dalton et al., 2020; Xue et al., 2025).

### 3.3 Main contributors

#### 3.3.1 Country

**Figure 3:**
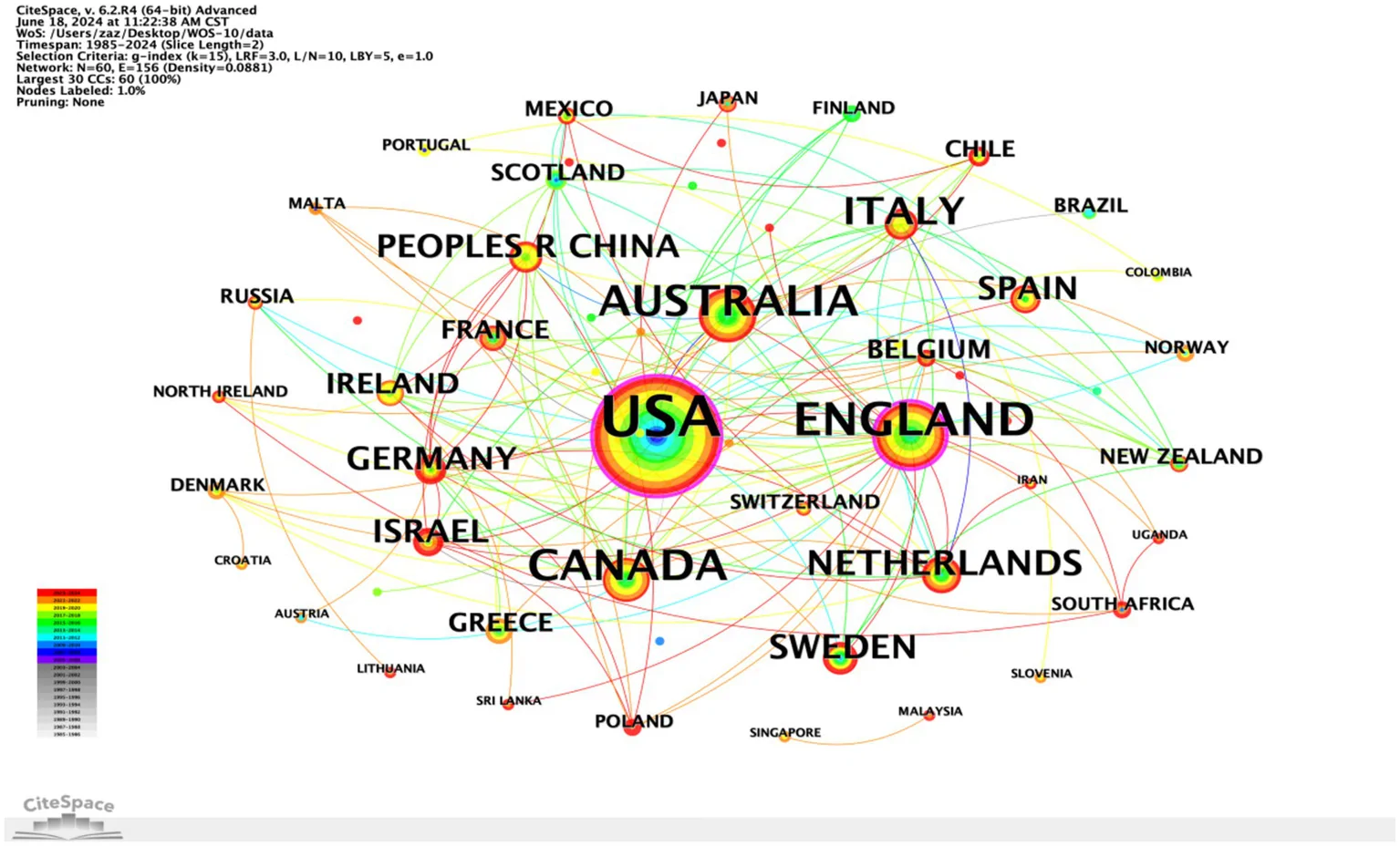
Country collaboration network.

The country collaboration network shows that research on narrative development in children with speech, language, and communication difficulties involved contributors from several regions. The USA had collaborative links with England, Canada, Australia, and a number of European countries, indicating its broad participation in cross-national research within the dataset. England and Australia were also connected with researchers from other English-speaking and European countries, including the Netherlands, Germany, and Sweden. Collaborative links were additionally observed among several European countries, such as Italy, Spain, Germany, and the Netherlands. China and Spain also appeared in the network, although their visible collaborative connections were comparatively limited. Overall, Figure 3 presents a geographically distributed collaboration network involving English-speaking, European, and Asian countries. These network links indicate observable international collaboration within the retrieved publications, but they should not be interpreted as evidence of national leadership, network centrality, or an increase in collaboration over time.

The publication counts of the 10 most productive countries further illustrate the geographic distribution of research output. The USA ranked first, with 280 publications since 1985, followed by England with 89 publications since 1998. Australia and Canada contributed 61 and 59 publications, respectively, while Italy produced 38 publications. The Netherlands, Sweden, China, Spain, and Germany also appeared among the 10 most productive countries, with publication counts ranging from 19 to 26. China and Spain appeared in the retrieved literature later than several other countries, with their first publications appearing in 2010 and 2011, respectively. These findings identify countries with relatively high levels of publication output during the study period. However, publication counts reflect research productivity rather than scholarly influence or leadership, while the current collaboration network provides a descriptive representation of cross-national links rather than evidence of changes in international collaboration over time, as shown in Table 1.

**Table 1:** Top 10 research countries in the field.

Rank	Country	Frequency	Year
1	USA	280	1985
2	ENGLAND	89	1998
3	AUSTRALIA	61	1997
4	CANADA	59	1992
5	ITALY	38	2005
6	NETHERLANDS	26	1991
7	SWEDEN	22	2000
8	CHINA	19	2010
9	SPAIN	19	2011
10	GERMANY	19	2002

#### 3.3.2 Institutions

**Figure 4:**
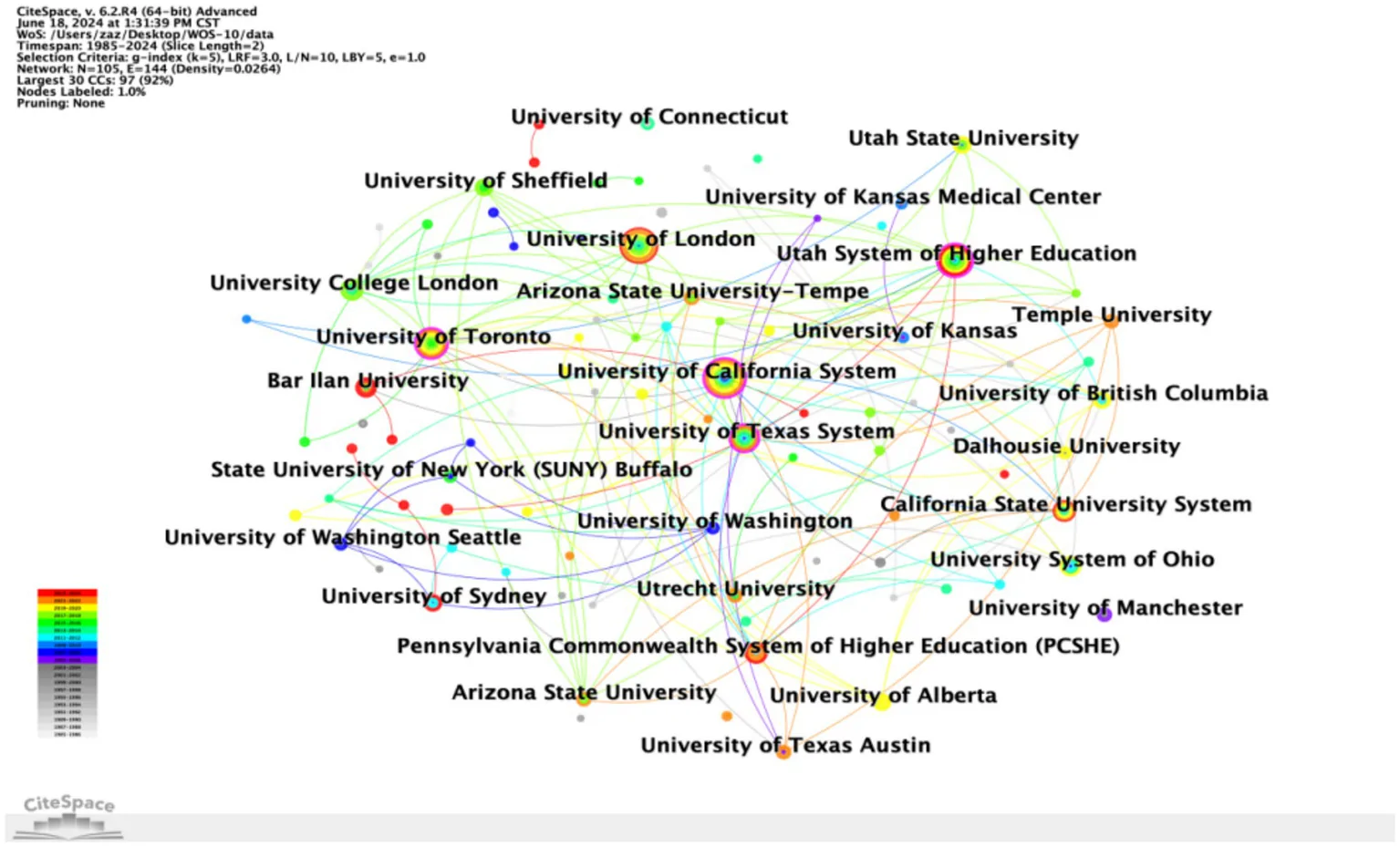
Institutional collaboration network.

The institutional collaboration network shows that research on narrative development in children with speech, language, and communication difficulties involved universities and higher education systems from North America, Europe, and other regions. The University of London and the University of California System displayed collaborative links with several institutions, including the University of Toronto, University College London, and the Utah System of Higher Education. Connections were also observed among institutions such as Bar Ilan University, the University of Sheffield, and the University of Washington. These links indicate that part of the research in the retrieved literature was conducted through cross-institutional and cross-regional collaboration. Overall, Figure 4 presents a distributed institutional network involving multiple universities and university systems. However, the visible network positions and collaboration links should be interpreted descriptively and do not independently demonstrate that particular institutions functioned as central hubs, formed the backbone of the field, or exercised greater scholarly influence.

In terms of publication frequency, the University of London and the University of California System ranked first, with 25 publications each, and their earliest publications in the dataset appeared in 2010 and 2004, respectively. The Utah System of Higher Education followed with 21 publications since 2009. Other productive institutions included the University of Toronto with 19 publications and the University of Texas System with 17 publications. University College London and Bar Ilan University contributed 12 and 11 publications, respectively, while the California State University System and the University System of Ohio each produced more than 10 publications. These results identify institutions with relatively high publication output in the retrieved dataset. Publication frequency reflects research productivity rather than institutional leadership or influence, and the collaboration links shown in Figure 4 describe observable relationships rather than formally tested network centrality, as shown in Table 2.

**Table 2:** Top 10 institutions in the field.

Rank	Institution	Frequency	Year
1	University of London	25	2010
2	University of California System	25	2004
3	Utah System of Higher Education	21	2009
4	University of Toronto	19	2001
5	University of Texas System	17	1998
6	California State University System	14	2000
7	University College London	12	2010
8	Bar Ilan University	11	2001
9	University System of Ohio	11	1994
10	Pennsylvania Commonwealth System of Higher Education (PCSHE)	11	2011

#### 3.3.3 Authors

**Figure 5:**
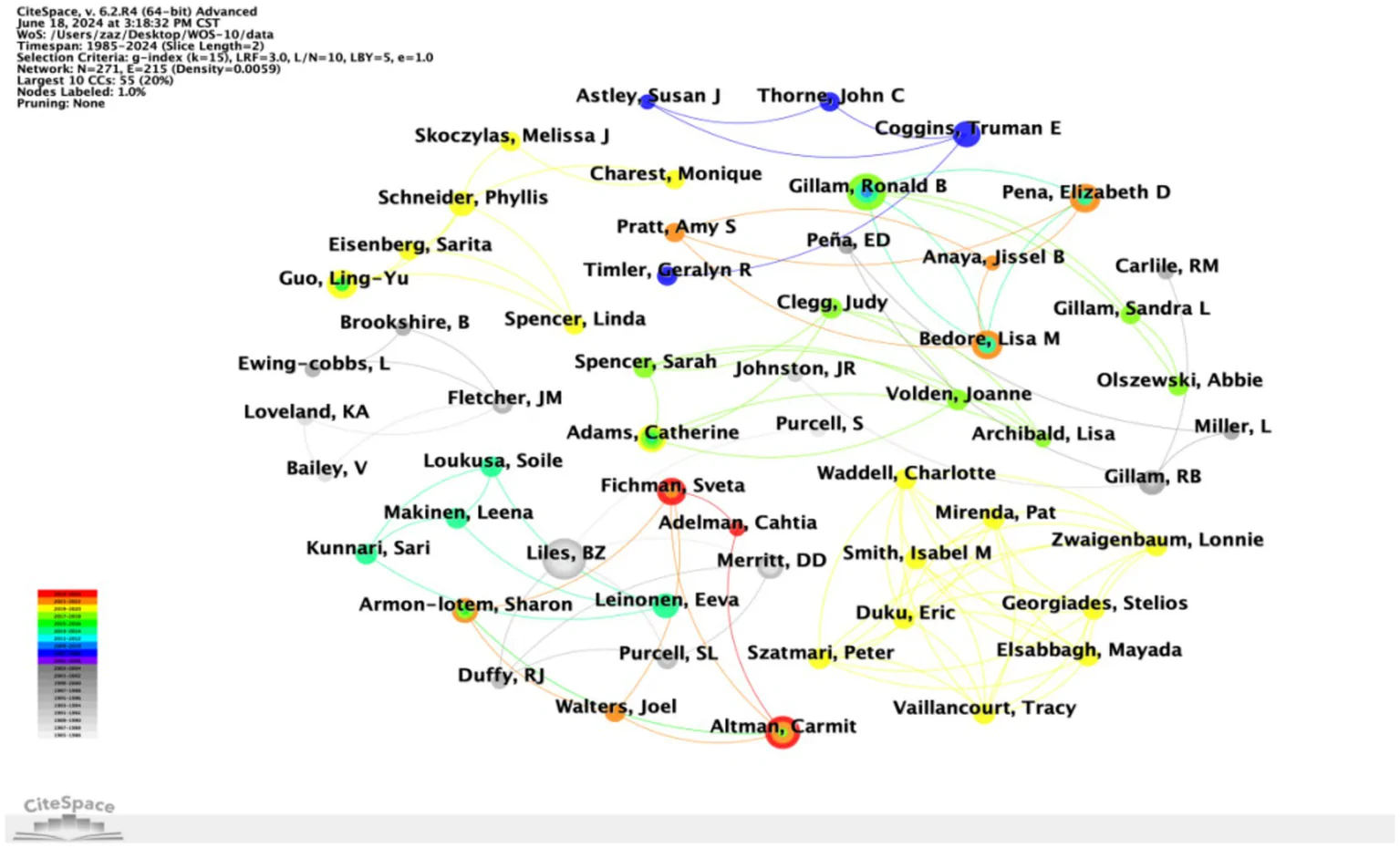
Author collaboration network.

The author collaboration network shows co-authorship relationships among researchers contributing to research on narrative development in children with speech, language, and communication difficulties. Liles, B. Z. and Gillam, Ronald B. were connected with several authors in the network, including Bedore, Lisa M. and Peña, Elizabeth D. Collaborative links were also observed for authors such as Altman, Carmit and Guo, Ling-Yu. These connections indicate that some publications were produced through collaboration among researchers from different institutions and regions. The network also includes authors from North America, Europe, and other areas, reflecting the geographic diversity of contributors represented in the dataset. Overall, Figure 5 provides a descriptive representation of co-authorship patterns within the retrieved literature. However, the number and visual position of collaborative links should not be interpreted as direct evidence that particular authors acted as central figures, key connectors, or leaders in shaping the field, because formal network centrality and longitudinal collaboration analyses were not conducted.

In terms of publication frequency, Liles, B. Z. ranked first with nine publications, with the earliest publication in the dataset appearing in 1985. Gillam, Ronald B. followed with eight publications, beginning in 2009, while Altman, Carmit contributed six publications from 2016 onward. Bedore, Lisa M. and Peña, Elizabeth D. each contributed between four and five publications. Fichman, Sveta, Adams, Catherine, and Guo, Ling-Yu also appeared among the 10 most productive authors in the retrieved literature. Some of these authors were linked through co-authorship, indicating participation in collaborative research. These findings identify authors with relatively high publication output and visible collaborative relationships within the dataset. Publication frequency reflects research productivity rather than scholarly impact or influence, and co-authorship links alone do not establish that particular authors shaped the research landscape, gained prominence over time, or drove the development of the field, as shown in Table 3.

**Table 3:** The most productive authors.

Rank	Author	Frequency	Year
1	Liles, BZ	9	1985
2	Gillam, Ronald B	8	2009
3	Altman, Carmit	6	2016
4	Bedore, Lisa M	5	2010
5	Fichman, Sveta	4	2021
6	Pena, Elizabeth D	4	2014
7	Adams, Catherine	4	2012
8	Guo, Ling-Yu	4	2016
9	Armon-lotem, Sharon	3	2016
10	Andreou, Maria	3	2016

### 3.4 Research hotspots

The keyword co-occurrence map reflects the main themes and research hotspots in research on narrative development in children with speech, language, and communication difficulties. The central keywords, such as “children” (100), “impairment” (100), and “ability” (96), indicate that the core focus of the research is on understanding the nature and extent of language and communication challenges in children. Other high-frequency terms, such as “language impairment” (88), “skills” (87), and “speech” (65), suggest a strong interest in how these impairments affect children’s language development and their ability to communicate effectively. Keywords like “discourse” (63), “adolescents” (57), and “comprehension” (56) highlight the importance of analyzing narrative skills and understanding language in both younger children and adolescents. The term “acquisition” (52) points to research exploring how children acquire language skills, even in the presence of impairments (as shown in Table 4).

**Table 4:** Research hotspots.

Rank	Keyword	Frequency	Year
1	Children	100	1991
2	Impairment	100	2001
3	Ability	96	1992
4	Language impairment	88	1999
5	Skills	87	1991
6	Speech	65	1991
7	Discourse	63	1994
8	Adolescents	57	1998
9	Comprehension	56	1991
10	Acquisition	52	2000

**Figure 6:**
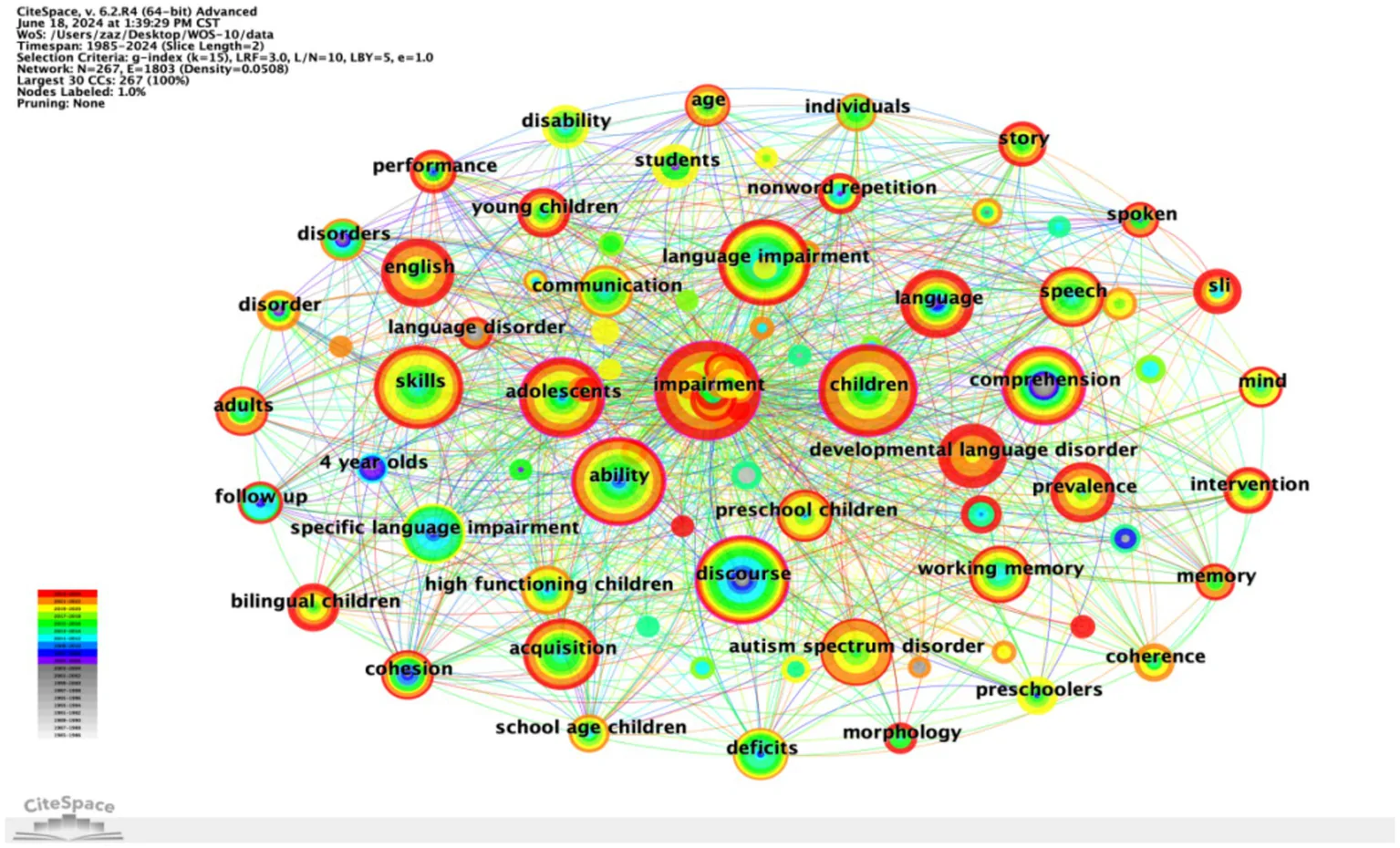
Research hotspots.

Research hotspots can be identified around language impairment, speech, comprehension, and acquisition. These areas reflect the focus on diagnosing, understanding, and intervening in speech and language disorders to support children’s overall linguistic and cognitive development. The prominence of these keywords suggests ongoing efforts to improve communication abilities through targeted interventions. The term “SLI” appeared frequently in the literature from the late 20th century to the early 21st century. However, with the growing movement toward terminological standardization in the field, “DLD” has gradually replaced “SLI” as the dominant term (Bishop et al., 2017; Xue et al., 2022). Meanwhile, research related to “ASD” has increased markedly since 2010, which reflects a shift in research perspectives from traditional structural language impairments to broader neurodevelopmental disorders that encompass social-pragmatic difficulties. Moreover, the co-occurrence patterns between diagnostic terms and research topics (e.g., “discourse,” “executive functions”) reveal distinct language dimensions emphasized across different disorder types (as shown in Figure 6).

#### 3.4.1 Language impairment and storytelling

In the context of language impairment, storytelling and retelling serve as powerful tools for both assessment and intervention. Research focuses on how children with DLD or SLI struggle with narrative tasks (Hessling and Schuele, 2020; Jensen de López et al., 2022). These children often face difficulties in organizing their thoughts, structuring coherent stories, and using appropriate linguistic markers, such as conjunctions and time expressions, to sequence events (Veraksa et al., 2020). Through storytelling, researchers can observe these deficits in real time, identifying specific language gaps, such as limited vocabulary or syntactic errors (Hettiarachchi et al., 2022; Hunt et al., 2022). Retelling tasks, where children recount a previously heard or read story, provide insights into their comprehension and memory skills (Gillon et al., 2023). Interventions based on storytelling typically aim to improve narrative coherence and linguistic complexity by providing structured support, such as story scaffolding or guided narrative building (Abi Zeid Daou et al., 2022; Favot et al., 2021; Pauls and Archibald, 2021). These narrative-based approaches have been shown to enhance not only storytelling abilities but also overall language skills, offering children a practical way to practice and internalize linguistic structures in a meaningful context (McCall et al., 2021).

#### 3.4.2 Speech and narrative development

Speech impairments, such as articulation issues, fluency disorders like stuttering, or phonological delays, significantly impact children’s ability to produce coherent and fluent narratives (Egbe et al., 2023). In storytelling, these impairments may manifest as disjointed speech, difficulty pronouncing words clearly, or hesitations that disrupt the flow of the story (Abu Talib et al., 2020). Research explores how speech impairments hinder the natural rhythm and clarity necessary for effective storytelling. Retelling exercises, where children recount familiar narratives, provide a framework to assess how speech difficulties interfere with narrative production, such as maintaining a clear sequence of events or expressing characters’ actions and emotions accurately (Hettiarachchi et al., 2022). Interventions often focus on integrating storytelling with speech therapy techniques to improve speech clarity and fluency (Estévez et al., 2021). For example, children may practice storytelling with speech prompts or visual supports to encourage smoother, more articulate speech. By combining narrative tasks with speech interventions, researchers aim to improve not only the mechanics of speech but also the children’s confidence and ability to communicate more effectively through stories (Veraksa et al., 2020).

#### 3.4.3 Comprehension and narrative understanding

Children with comprehension difficulties often struggle with understanding and interpreting narratives, particularly when stories involve complex plots, multiple characters, or abstract themes (Kelso et al., 2022). Research focuses on how children with language impairments, such as DLD, process and make sense of stories (McGregor, 2020). Storytelling and retelling are used as assessment tools to gauge how well children understand narrative structures, infer meaning from context, and interpret the motivations of characters (Boyd et al., 2020). For children with comprehension issues, the challenge lies in following the logical sequence of events and connecting the narrative details to form a coherent understanding. Interventions aimed at improving comprehension often involve breaking down stories into simpler, manageable components, using visual aids, or interactive storytelling methods (Tarvainen et al., 2020). These techniques help children engage more deeply with the narrative, enhancing their ability to understand and recall important details. Over time, narrative-based interventions strengthen not only comprehension but also critical thinking and inferential reasoning, which are essential for broader academic success (Capodieci et al., 2020).

#### 3.4.4 Acquisition and narrative skills

Language acquisition is closely linked to the development of narrative skills, with storytelling serving as a key marker of linguistic growth (Kidd and Garcia, 2022). For typically developing children, storytelling evolves naturally as they acquire vocabulary, grammar, and the ability to structure coherent narratives. However, for children with language impairments, research shows that their ability to construct and retell stories may lag behind, often characterized by less complex sentence structures, limited use of cohesive devices, and difficulty organizing story elements logically (Erdemir and Brutt-Griffler, 2022). Storytelling and retelling are frequently used in studies to track how these children acquire and use new language forms in real time (Scott et al., 2022). Interventions targeting language acquisition often involve narrative-based tasks where children practice storytelling in structured settings, with guidance on vocabulary use, sentence formation, and story sequencing (Pico et al., 2021). These tasks help children with impairments internalize language rules and improve their ability to construct more complex and coherent narratives over time. The use of narrative frameworks accelerates language acquisition by providing practical, meaningful contexts in which children can actively apply their developing language skills (Kendeou et al., 2020).

### 3.5 Research frontiers

**Figure 7:**
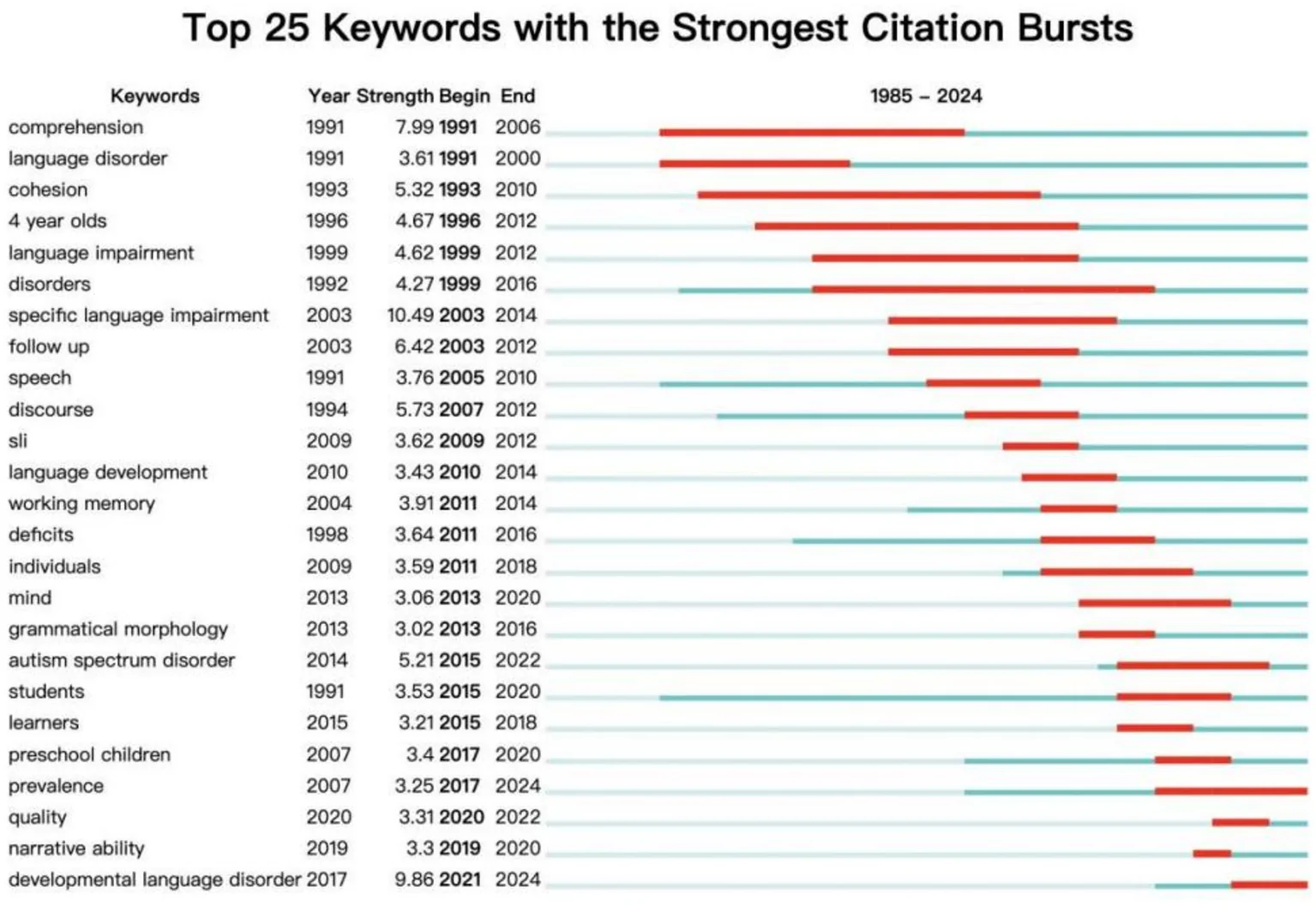
Research frontiers.

Figure 7 presents the 25 keywords with the strongest citation bursts between 1985 and 2024, illustrating changes in the topics receiving concentrated scholarly attention over time. Earlier bursts included comprehension (1991–2006), language disorder (1991–2000), and cohesion (1993–2010), indicating sustained attention to language comprehension, impairment, and narrative organization. Subsequent bursts involved more specific topics, including autism spectrum disorder (2015–2022), grammatical morphology (2013–2016), students (2015–2020), and learners (2015–2018). Among the most recent keywords, prevalence and developmental language disorder both showed bursts extending from 2021 to 2024. Developmental language disorder had a comparatively strong burst value of 9.86, whereas prevalence had a burst value of 2.35. These two terms therefore represent the most recent areas of bibliometric prominence in the dataset. In this section, research frontiers refer to topics showing recent citation-burst activity rather than confirmed clinical advances, diagnostic effectiveness, or epidemiological findings.

#### 3.5.1 Prevalence-related research

The keyword prevalence showed a citation burst from 2021 to 2024, indicating recent scholarly attention to the occurrence, identification, and distribution of childhood language difficulties. This bibliometric pattern does not provide an estimate of prevalence itself, but instead shows that prevalence-related questions became more visible in the recent literature. Within this research context, storytelling and retelling tasks have been examined as possible sources of information about children’s communication and narrative performance (Ralli et al., 2021). Such tasks allow researchers to observe story organization, event sequencing, vocabulary use, grammatical structure, and narrative coherence (Khan et al., 2021; Winters et al., 2022). They have also been discussed in studies concerned with identifying language difficulties across different groups and educational contexts (Adlof, 2020). However, the keyword burst does not establish that narrative tasks independently determine prevalence or function as validated diagnostic instruments. Rather, the supporting literature helps contextualize why assessment and identification may be associated with the recent prominence of prevalence-related research (Hernández-Torrano and Courtney, 2021).

#### 3.5.2 Developmental language disorder

Developmental language disorder showed a strong citation burst from 2021 to 2024, indicating that it became a prominent topic in the most recent literature included in the analysis. Studies associated with this topic have frequently examined children’s narrative organization, coherence, vocabulary, grammatical structures, and use of temporal and causal connections (Veraksa et al., 2020). Storytelling and retelling tasks have been used to observe how children with DLD comprehend, organize, and reproduce narrative information (Bowles et al., 2020; Freed and Cain, 2021). Narrative-based activities have also been explored as possible forms of instructional or therapeutic support for expressive and receptive language development (Dobinson and Dockrell, 2021). Structured storytelling may provide opportunities to practice sequencing, sentence construction, and coherent expression (Danaei et al., 2020). Nevertheless, the citation-burst analysis indicates recent research attention rather than demonstrating the diagnostic validity of storytelling tasks or the effectiveness of particular interventions. The cited studies therefore provide contextual interpretation of the burst keyword rather than additional bibliometric evidence (Brodin and Renblad, 2020).

## 4 Conclusion

This study used CiteSpace to examine the development of research on narrative and storytelling among children with speech, language, and communication difficulties from 1985 to 2024. The findings showed a general increase in publication output and identified the countries, institutions, and authors with relatively high levels of productivity and observable collaboration. CiteSpace generated seven keyword clusters, which were subsequently synthesized by the authors into four broader research themes: Language and Communication Disorders, Cognitive and Social Dimensions of Communication, Medical and Developmental Perspectives, and Speech Rate and Communication Fluency. The citation burst analysis further indicated that prevalence and developmental language disorder were the most recent topics of bibliometric prominence between 2021 and 2024. Overall, the study provides a structured overview of the publication trajectory, collaboration patterns, thematic organization, and recent areas of scholarly attention in this field. These findings clarify how research interests have evolved over time while distinguishing direct bibliometric outputs from the authors’ higher-order thematic interpretation of the retrieved literature.

The findings offer several practical implications, although these should be interpreted cautiously. For researchers, the identified clusters and recent burst keywords may help locate established areas of inquiry and topics that require further investigation. For educators and speech and language professionals, the prominence of narrative organization, social cognition, developmental language disorder, and communication fluency may provide a useful reference when selecting issues for future assessment, teaching, or intervention research. The country, institutional, and author analyses may also support the identification of potential collaborators and research networks. However, the present bibliometric evidence does not establish the diagnostic validity of storytelling tasks, the effectiveness of narrative-based interventions, or their effects on curriculum development, therapeutic outcomes, or policy implementation. Therefore, the results should be used to inform future empirical studies rather than as direct guidance for clinical or educational decision-making. Further experimental, longitudinal, and practice-based research is needed before stronger applied conclusions can be drawn in these contexts.

Several limitations should be acknowledged. First, the analysis relied exclusively on the Web of Science Core Collection, which may have excluded relevant publications indexed elsewhere. Future studies should integrate additional databases to improve coverage and compare findings across sources. Second, the cluster labels generated by CiteSpace and the four broader themes synthesized from them involve interpretive judgment, while keyword-based analyses may be affected by synonyms, inconsistent terminology, and low-frequency concepts. Future research should apply transparent coding procedures and conduct sensitivity checks to examine alternative thematic structures. Third, the study used CiteSpace only and did not cross validate the networks with VOSviewer or other bibliometric tools. Citation bursts, co-occurrence links, and collaboration patterns also indicate scholarly attention and association rather than intervention effectiveness or clinical impact. Future investigations should combine multiple bibliometric platforms and complement bibliometric mapping with systematic reviews, meta-analyses, or empirical studies to assess the robustness, practical relevance, and clinical implications of the identified research patterns.
